# Current Tobacco/Nicotine Use and Pelvic Pain and Urgency/Frequency (PUF)-Defined Urinary Symptom Burden Among Lebanese University-Aged Adults: A Cross-Sectional Study

**DOI:** 10.7759/cureus.112626

**Published:** 2026-07-13

**Authors:** Aoumar G Chamma, Wendy Saliba, Anthony Nasr, Linda Chamma

**Affiliations:** 1 Cardiology, University of Balamand, Beirut, LBN; 2 Radiology, American University of Beirut Medical Center, Beirut, LBN; 3 Urology, Lebanese University, Beirut, LBN

**Keywords:** caffeine, interstitial cystitis/bladder pain syndrome, lebanon, lower urinary tract symptoms, narghile, puf score, smoking, young adults

## Abstract

Background

Smoking has been linked to several urological conditions, including lower urinary tract symptoms and interstitial cystitis/bladder pain syndrome (IC/BPS). Cigarette and narghile use remains common among young adults in Lebanon. This study assessed the association between current tobacco/nicotine use and pelvic pain and urgency/frequency (PUF)-defined urinary symptom burden among Lebanese students and recent graduates.

Methods

This cross-sectional analytical study included 476 Lebanese university students and recent graduates aged 18-30 years during the 2019-2020 academic year. Urinary symptoms were assessed using the PUF questionnaire. A PUF score >2 was analyzed as low-threshold PUF screening-positive urinary symptom burden, and a PUF score of >9 was analyzed as higher-threshold PUF screening-positive symptom burden. Smoking exposure was assessed using items adapted from the Global Adult Tobacco Survey. Associations were assessed using chi-square testing, one-way analysis of variance with Welch correction when appropriate, crude prevalence ratios, multivariable logistic regression, and modified Poisson regression with robust error variance, both with current smoking as the primary exposure and adjusting for age, caffeine intake, sex, body mass index, and university type.

Results

The sample included 237 males (49.8%) and 239 females (50.2%), with a mean age of 23.57 (SD: 3.43) years and a mean body mass index of 24.11 (SD: 3.82) kg/m². Current smokers represented 167 participants (35.1%). Low-threshold PUF screening-positive urinary symptom burden (PUF >2) was present in 137 smokers (82.0%) and 56 non-smokers (18.1%) (χ²(1) = 183.70, p < 0.001; crude prevalence ratio: 4.53, 95% CI: 3.53-5.80). Higher-threshold PUF screening-positive symptom burden (PUF >9) was present in 67 smokers (40.1%) and 6 non-smokers (1.9%) (χ²(1) = 121.69, p < 0.001; crude prevalence ratio: 20.66, 95% CI: 9.16-46.62). In adjusted prevalence-ratio models, smoking remained associated with low-threshold PUF screening-positive urinary symptom burden (adjusted PR: 4.43, 95% CI: 3.49-5.63) and higher-threshold PUF screening-positive symptom burden (adjusted PR: 18.12, 95% CI: 8.29-39.64). Daily cigarette/cigar quantity was associated with a higher mean PUF score among 89 users (ANOVA F(3,85) = 10.14, p < 0.001) and with higher PUF >9 prevalence among the same 89 users with daily quantity data (χ²(3) = 11.38, p = 0.010).

Conclusions

Current tobacco/nicotine use was strongly associated with higher questionnaire-based PUF screening-positive urinary symptom burden among Lebanese university-aged adults. A dose-response pattern was observed among cigarette/cigar users. Because this was a cross-sectional questionnaire-based study without clinical confirmation, the findings should be interpreted as screening-based associations rather than confirmed clinical diagnoses.

## Introduction

Smoking remains a major preventable cause of morbidity and premature mortality worldwide [1]. In Lebanon, tobacco use among university students has been reported at high levels, with narghile use representing an important regional pattern of exposure [2]. Beyond its established association with malignancy and cardiovascular disease, tobacco exposure is relevant to urological health and has been linked to storage urinary symptoms, bladder pain, and broader lower urinary tract symptom (LUTS) burden [3,4].

LUTS include storage symptoms, such as urgency, frequency, nocturia, and urinary incontinence, as well as voiding symptoms, such as hesitancy and incomplete emptying [5]. Although LUTS are more often studied in older populations, young adults may also report clinically meaningful symptoms, and these symptoms may be underrecognized in routine clinical encounters [6,7].

Interstitial cystitis/bladder pain syndrome is characterized by chronic bladder-related pain or discomfort accompanied by urgency or frequency after exclusion of other causes. Diagnosis requires clinical evaluation and exclusion of urinary tract infection, stones, malignancy, gynecological disease, and other overlapping conditions [8-10]. The Pelvic Pain and Urgency/Frequency Questionnaire assesses urinary frequency, nocturia, urgency, pelvic pain, and symptom bother; it can support symptom screening, but it does not replace clinical diagnosis [11,12].

Several mechanisms may explain the relationship between smoking and urinary symptoms. Tobacco exposure can impair endothelial function, increase systemic inflammation, and injure urothelial cells. Experimental data also suggest a potential role for platelet-activating factor signaling and urothelial barrier disruption [13-17]. Caffeine may aggravate urgency and frequency through bladder stimulation and changes in detrusor activity [18-20].

Regional data on questionnaire-based PUF screening-positive urinary symptom burden among young Lebanese adults are limited. This study evaluated the association between current smoking and low-threshold PUF screening-positive urinary symptom burden (PUF >2) or higher-threshold PUF screening-positive symptom burden (PUF >9) among Lebanese students and recent graduates while exploring smoking modality, cumulative smoking exposure, daily cigarette/cigar quantity, and caffeine intake.

## Materials and methods

Study design and setting

This cross-sectional analytical study was conducted during the 2019-2020 academic year among Lebanese university students and recent graduates. The study is reported in line with relevant cross-sectional reporting principles from the Strengthening the Reporting of Observational Studies in Epidemiology (STROBE) statement [21].

Ethical approval

Ethical approval was obtained from the Institutional Review Board of Lebanese University. No approval number was assigned. Electronic informed consent was obtained from all participants before completion of the anonymous online questionnaire. Participation was voluntary, and no personal identifiers were collected.

Participants

The target population included Lebanese students and recent graduates aged 18-30 years from different academic disciplines, including medicine, pharmacy, dentistry, engineering, and sciences. Participants were recruited from public and private universities across Lebanon through social media and class representative networks. A non-probability convenience sampling strategy was used, with snowball distribution through university-linked social media groups, WhatsApp groups, and class representative networks. Because recruitment was not random, the findings should be interpreted as associations within the recruited university-aged sample rather than population-level prevalence estimates for all Lebanese young adults. Recent graduates were included because they belonged to the same age range and had recently been recruited through university-linked networks.

Eligible participants were aged 18-30 years, provided informed consent, and were either current smokers or never-smoking non-smokers. Current smokers were defined as participants reporting current use of at least one included tobacco or nicotine modality. Smoking modalities included manufactured cigarettes, hand-rolled cigarettes, narghile, vape, pipe, and cigars. Participants who denied both current and previous tobacco or nicotine use were classified as never-smoking non-smokers. Former smokers, defined as participants with any prior tobacco or nicotine use without current use, were excluded from the primary analytic comparison. No screened respondent met the former-smoker exclusion category.

Exclusion criteria were age below 18 or above 30 years, pregnancy, diabetes mellitus, current or chronic antibiotic use, structural urological abnormalities predisposing to reflux or recurrent urinary tract infection, use of diaphragm or spermicidal contraceptive methods, former smoking (any prior tobacco or nicotine use without current use), and refusal to provide informed consent. Of 505 initial responses, 476 participants were included after excluding 29 responses.

Responses were screened sequentially for eligibility. Participants were excluded if they did not provide consent, were outside the 18-30-year age range, were pregnant, had diabetes mellitus, reported current or chronic antibiotic use, reported structural urological abnormalities or excluded contraceptive methods, had incomplete key data, or were former smokers without current tobacco/nicotine use. The final analytic sample included 476 participants after exclusion of 29 responses.

Sample size

Sample size was estimated for comparison of two independent proportions with unequal exposed-to-unexposed allocation using the formula:

$n1 = {[Z&alpha;/2&radic;((1 + 1/r)P̄(1 &minus; P̄)) + Z&beta;&radic;(P1(1 &minus; P1) + P0(1 &minus; P0)/r)]&sup2;} / (P1 &minus; P0)&sup2;$

Here, n0 = r × n1, P1 was the expected screening-positive prevalence among smokers, P0 was the expected screening-positive prevalence among non-smokers, r was the non-smoker-to-smoker allocation ratio, and P̄ was the weighted pooled prevalence. The calculation used P0 = 3.5%, P1 = 10.5%, r ≈ 1.86, 95% confidence, and 80% power.

Sample size was calculated a priori using StatCalc for cross-sectional studies. The calculation assumed a smoking prevalence of approximately 35% based on previously reported tobacco use among Lebanese university students [2]. Because reliable local estimates of PUF screening-positive urinary symptom burden were unavailable, outcome prevalences of 3.5% among non-smokers and 10.5% among smokers were selected as planning assumptions, corresponding to an expected relative risk of 3. The assumed non-smoker-to-smoker allocation ratio was approximately 1.86, corresponding to approximately 150 smokers and 279 non-smokers. With 95% confidence and 80% power, the required sample was 429 participants. The final analytic sample exceeded this requirement and included 476 participants, comprising 167 smokers and 309 non-smokers. Observed low-threshold PUF screening-positive urinary symptom burden (PUF >2) was higher than assumed during sample-size planning, indicating that the a priori assumptions were conservative and that the final sample had considerably more power than the minimum requirement for the primary outcome.

Data collection and measures

Data were collected using a Google Forms (Alphabet Inc., Mountain View, CA) questionnaire distributed through Facebook, WhatsApp, class representatives, and social networks. Survey invitations were distributed to an estimated 1,120 eligible individuals through university-linked groups and representative networks. Because invitations were shared electronically, this denominator is an estimate rather than a direct individual count; the estimated response rate was 45.1% (505/1,120). Because invitations were distributed through group-based electronic channels, the denominator was estimated from the distribution networks rather than directly tracked at the individual level. Therefore, the response rate should be interpreted as approximate. The questionnaire was administered in English, which was the language selected for the study instrument and recruitment setting. No validated Arabic version of the PUF questionnaire was used in this study. The form included sociodemographic variables, smoking-related items adapted from the Global Adult Tobacco Survey [22], caffeine intake, and the PUF questionnaire [11].

Smoking exposure variables included current smoking status, main smoking modality, smoking duration, mixed-modality use, daily cigarette/cigar quantity, estimated cigarette/cigar pack-years, narghile sessions per week, vape frequency, and nicotine-containing vape use. Daily quantity analysis was restricted to cigarette/cigar users because cigarette counts could not be converted reliably into equivalent narghile or vape exposure.

The PUF questionnaire includes eight items assessing urinary frequency, nocturia, urgency, pelvic pain, and pain during or after sexual intercourse. Total scores range from 0 to 35 [11-13]. For this study, the intercourse-related pain item was coded as 0 for participants reporting no sexual activity, according to the predefined study scoring approach. A PUF score >2 was used as a study-defined low-threshold marker of questionnaire-reported urinary symptom burden, while a PUF score of >9 was used as a study-defined higher-threshold marker of symptom burden. These thresholds were not treated as validated diagnostic cutoffs. No clinical diagnosis of IC/BPS or another urological disorder was assigned because the study did not include urinalysis, urine culture, cystoscopy, urodynamic testing, confirmation of symptom chronicity, or specialist evaluation.

Caffeine intake was recorded as use versus non-use. Dose, beverage type, timing, energy drink use, and daily caffeine amount were not available.

Statistical analysis

Data were analyzed using Statistical Product and Service Solutions (SPSS, version 19.0; IBM SPSS Statistics for Windows, Armonk, NY). Figures were created using Microsoft PowerPoint for Microsoft 365 (Microsoft Corporation, Redmond, WA) and exported as high-resolution PNG images. Continuous variables were reported as mean (standard deviation), and categorical variables were reported as n (%). Associations between categorical variables were assessed using Pearson's chi-square testing. Continuous variables were compared using independent-samples t-tests. Mean PUF scores across smoking modalities and daily cigarette/cigar quantity groups were compared using one-way analysis of variance (ANOVA). Welch's correction was used when variances were unequal. Tukey's honest significant difference post-hoc testing was used for daily cigarette/cigar quantity comparisons because Levene's test was not significant. Multivariable models were built using current smoking as the primary exposure. Covariates were selected a priori based on clinical plausibility and prior literature and were not selected according to statistical significance in univariable testing. Age, sex, and BMI were included as demographic and clinical covariates. Caffeine intake was included because caffeine may influence urinary urgency and frequency and may differ according to smoking behavior [18-20]. University type was included as a proxy for differences in socioeconomic and academic environment. Caffeine was coded as use versus non-use because dose, beverage type, timing, energy drink use, and daily caffeine amount were not available. Logistic regression was used to estimate adjusted odds ratios. Because low-threshold PUF screening-positive urinary symptom burden (PUF >2) was common, odds ratios were interpreted as association estimates rather than approximations of relative risk. Modified Poisson regression with robust error variance was therefore used to estimate adjusted prevalence ratios, which are more directly interpretable for common binary outcomes in cross-sectional studies [23]. The modified Poisson models were fitted using the generalized linear (GENLIN) procedure in IBM SPSS Statistics (version 19.0), with a Poisson distribution, log link function, and robust covariance matrix estimator (COVB=ROBUST). Sparse outcome events were considered when interpreting the higher-threshold PUF screening-positive symptom burden (PUF >9) models, especially because only six non-smokers met the PUF >9 threshold. Exploratory modality-stratified sensitivity analyses were performed using modified Poisson regression with robust error variance. Three exposure definitions were evaluated separately: any current tobacco/nicotine use versus never-smoking non-use, cigarette/cigar-only use versus never-smoking non-use, and narghile-only use versus never-smoking non-use. Each model adjusted for age, caffeine intake, sex, body mass index, and university type. Mixed-modality users were included only in the any-use model and excluded from the cigarette/cigar-only and narghile-only models. A two-sided p-value <0.05 was considered statistically significant.

## Results

Participant flow and baseline characteristics

**Figure 1:**
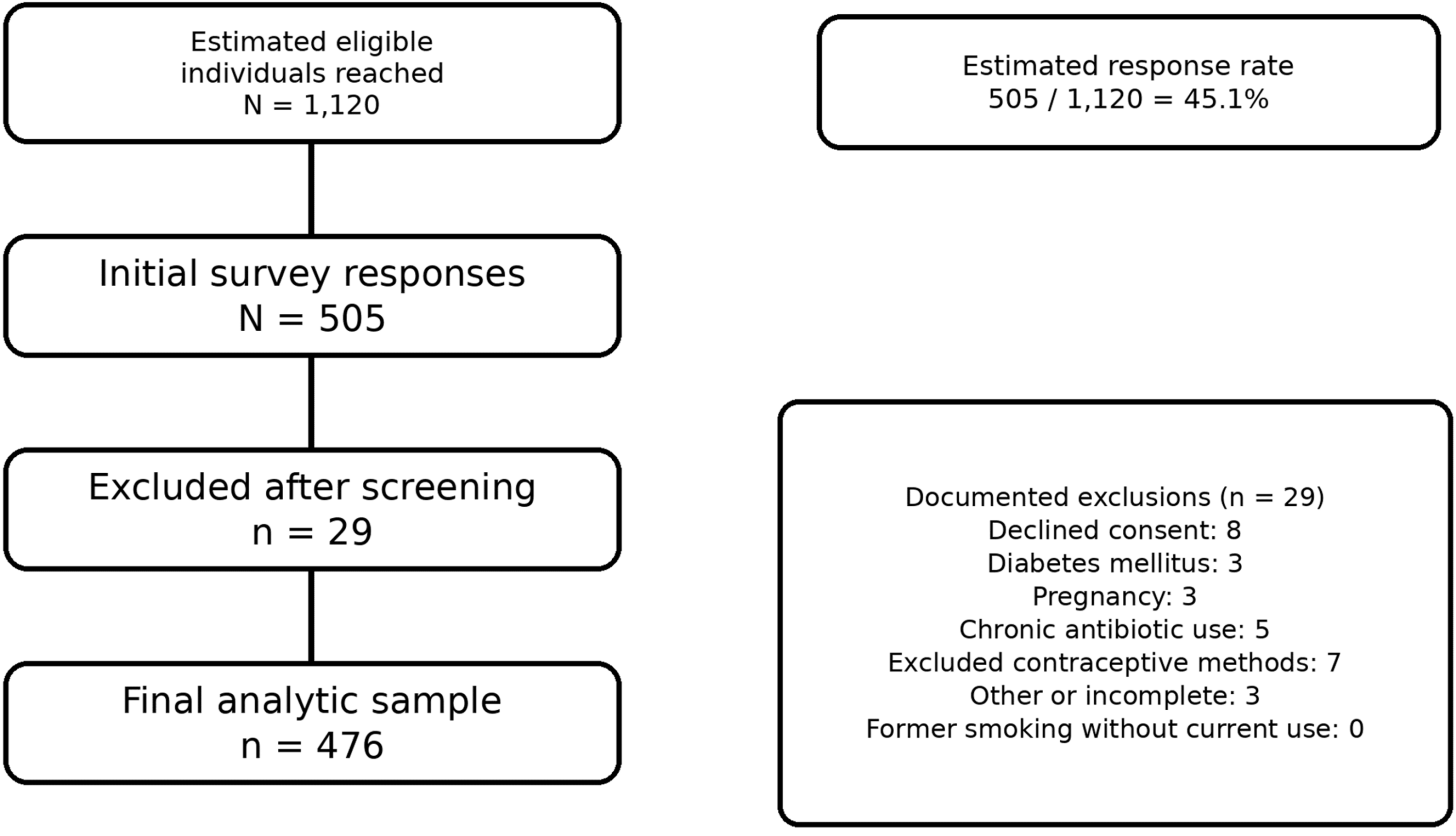
Participant flow diagram. The diagram shows the estimated number of eligible individuals reached, responses received, excluded responses, and the final analytic sample. Figures created using Microsoft PowerPoint for Microsoft 365 (Microsoft Corporation, Redmond, WA) and exported as a high-resolution PNG image.

Survey invitations reached an estimated 1,120 eligible individuals, and 505 responses were received, yielding an estimated response rate of 45.1%. Twenty-nine responses were excluded, leaving 476 participants for analysis. The sample included 237 males (49.8%) and 239 females (50.2%), with a mean age of 23.57 (SD: 3.43) years and a mean body mass index of 24.11 (SD: 3.82) kg/m². Current smokers represented 167 participants (35.1%), and non-smokers represented 309 participants (64.9%). Caffeine use was reported by 400 participants (84.0%). Public university students represented 245 participants (51.5%), and private university students represented 231 participants (48.5%). Participant flow is shown in Figure 1, and baseline characteristics stratified by smoking status are summarized in Table 1.

**Table 1:** Baseline characteristics stratified by smoking status. BMI: body mass index; SD: standard deviation Continuous variables are presented as mean (SD) and were compared using independent-samples t-tests. Categorical variables are presented as n (%) and were compared using Pearson's chi-square tests. P-values compare current smokers with never-smoking nonsmokers.

Characteristic	Smokers (n = 167)	Non-smokers (n = 309)	Total (N = 476)	p-value
Age, mean (SD), years	23.72 (3.39)	23.49 (3.45)	23.57 (3.43)	0.483
BMI, mean (SD), kg/m²	24.31 (3.91)	24.00 (3.77)	24.11 (3.82)	0.404
Male sex	94 (56.3%)	143 (46.3%)	237 (49.8%)	0.037
Female sex	73 (43.7%)	166 (53.7%)	239 (50.2%)	
Caffeine users	148 (88.6%)	252 (81.6%)	400 (84.0%)	0.044
Non-caffeine users	19 (11.4%)	57 (18.4%)	76 (16.0%)	
Public university	86 (51.5%)	159 (51.5%)	245 (51.5%)	0.993
Private university	81 (48.5%)	150 (48.5%)	231 (48.5%)	
Mount Lebanon	48 (28.7%)	91 (29.4%)	139 (29.2%)	0.955
North Lebanon	43 (25.7%)	69 (22.3%)	112 (23.5%)	
Beirut	29 (17.4%)	51 (16.5%)	80 (16.8%)	
Bekaa	17 (10.2%)	35 (11.3%)	52 (10.9%)	
South Lebanon	18 (10.8%)	39 (12.6%)	57 (12.0%)	
Nabatieh	12 (7.2%)	24 (7.8%)	36 (7.6%)	
Faculty of Medicine	36 (21.6%)	80 (25.9%)	116 (24.4%)	0.842
Faculty of Science	25 (15.0%)	45 (14.6%)	70 (14.7%)	
Faculty of Pharmacy	27 (16.2%)	40 (12.9%)	67 (14.1%)	
Faculty of Engineering	33 (19.8%)	56 (18.1%)	89 (18.7%)	
Faculty of Dentistry	17 (10.2%)	29 (9.4%)	46 (9.7%)	
Other faculties	29 (17.4%)	59 (19.1%)	88 (18.5%)	

Smoking exposure profile

Among the 167 current smokers, median smoking duration was four years (interquartile range: 2-6 years), and 49 participants (29.3%) reported mixed-modality use. Among the 89 cigarette/cigar users with daily quantity data, the mean estimated cigarette/cigar exposure was 3.2 (SD: 2.6) pack-years. The most common narghile exposure frequencies were one to two sessions per week (43.9%) and three to four sessions per week (37.9%). Smoking exposure patterns among current smokers are summarized in Table 2.

**Table 2:** Smoking exposure profile among current smokers. SD: standard deviation Values are presented as n (%) unless otherwise stated. Percentages were calculated among current smokers unless a modality-specific denominator is shown.

Smoking exposure variable	Value
Smoking duration <1 year	21 (12.6%)
Smoking duration 1-3 years	56 (33.5%)
Smoking duration 4-5 years	45 (26.9%)
Smoking duration >5 years	45 (26.9%)
Mixed-modality tobacco/nicotine use	49 (29.3%)
Single-modality tobacco/nicotine use	118 (70.7%)
Cigarette/cigar users with daily quantity data	89 (53.3%)
Estimated pack-years among cigarette/cigar users, mean (SD)	3.2 (2.6)
Narghile users reporting 1-2 sessions/week	29/66 (43.9%)
Narghile users reporting 3-4 sessions/week	25/66 (37.9%)
Narghile users reporting >=5 sessions/week	12/66 (18.2%)
Vape users reporting daily use	5/8 (62.5%)
Vape users reporting nicotine-containing products	7/8 (87.5%)

Smoking status and PUF screening-positive urinary symptom burden

**Figure 2:**
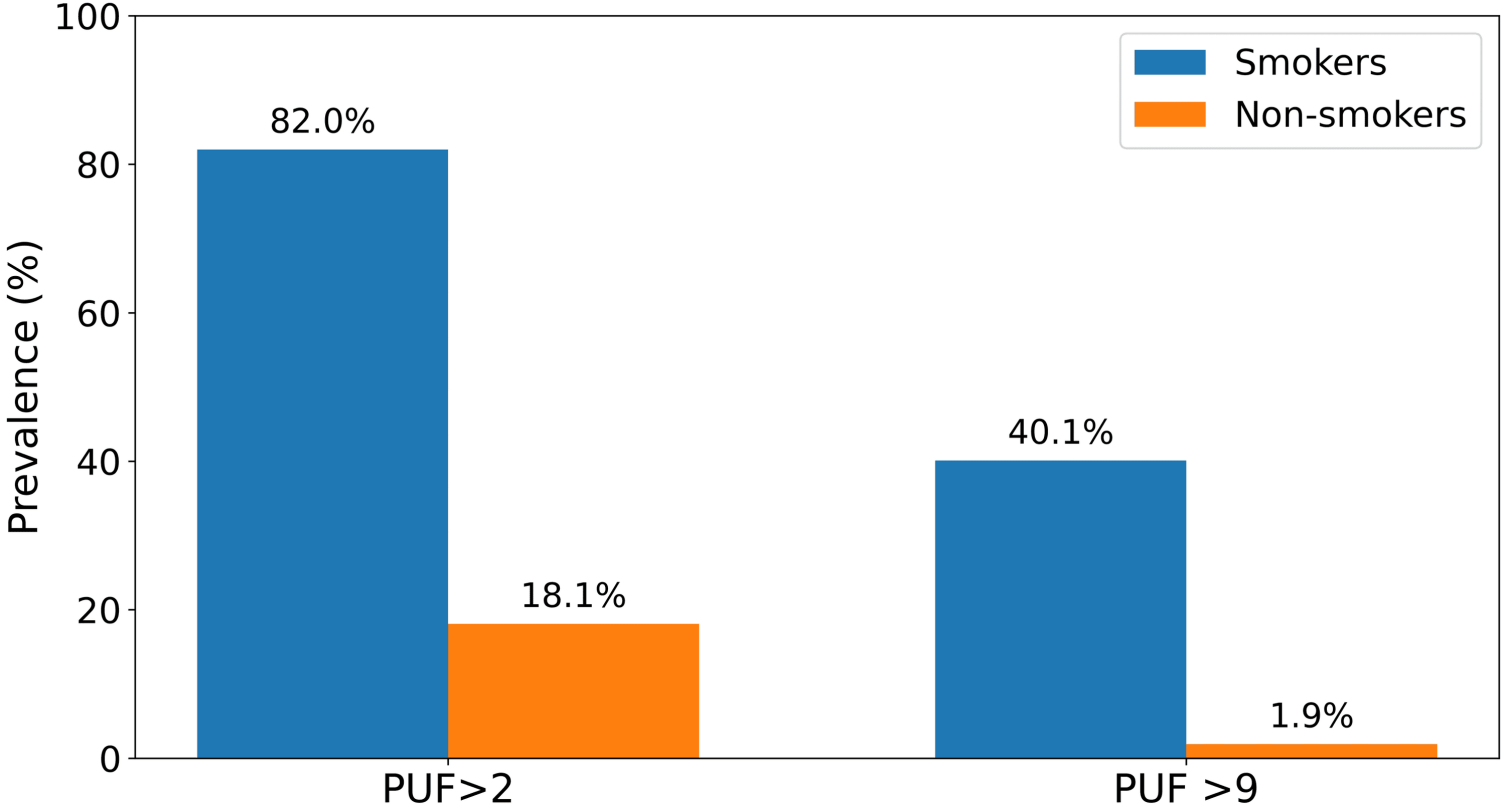
Prevalence of PUF screening-positive urinary symptom burden by smoking status. Bars show the prevalence of pelvic pain and urgency/frequency (PUF) screening-positive urinary symptom burden (PUF >2) and higher-threshold PUF screening-positive symptom burden (PUF >9) among current smokers and never-smoking non-smokers.

Smoking was strongly associated with low-threshold PUF screening-positive urinary symptom burden (PUF >2). Low-threshold PUF screening-positive urinary symptom burden (PUF >2) was present in 137 smokers (82.0%) and 56 non-smokers (18.1%) (χ²(1) = 183.70, p < 0.001). The crude prevalence ratio was 4.53 (95% CI: 3.53-5.80). PUF >9 was present in 67 smokers (40.1%) and 6 non-smokers (1.9%) (χ²(1) = 121.69, p < 0.001). The crude prevalence ratio was 20.66 (95% CI: 9.16-46.62). The association between smoking status and PUF screening-positive outcomes is summarized in Table 3 and Figure 2.

**Table 3:** Association between smoking status and PUF screening-positive urinary symptom burden. Association between smoking status and pelvic pain and urgency/frequency (PUF) screening-positive urinary symptom burden. Pearson's chi-square statistics: PUF >2, χ²(1) = 183.70; PUF >9, χ²(1) = 121.69.

Outcome	Smokers (n = 167)	Non-smokers (n = 309)	Crude prevalence ratio (95% CI)	p-value
Low-threshold PUF screening-positive urinary symptom burden (PUF >2)	137 (82.0%)	56 (18.1%)	4.53 (3.53-5.80)	<0.001
PUF >9	67 (40.1%)	6 (1.9%)	20.66 (9.16-46.62)	<0.001

Smoking modality and mean PUF score

**Figure 3:**
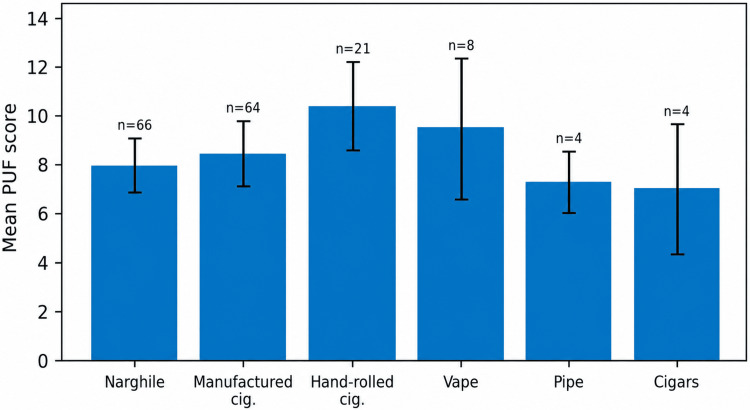
Mean PUF score by smoking modality. Bars show the mean pelvic pain and urgency/frequency (PUF) scores among current smokers according to main smoking modality. Error bars represent 95% confidence intervals.

All 167 smokers were included in the smoking-modality PUF-score analysis. Narghile and manufactured cigarettes were the most common smoking modalities. Mean PUF scores were elevated across smoking modalities, but modality-specific differences were not statistically significant (Levene F(5,161) = 2.53, p = 0.031; Welch F(5,17.3) = 1.59, p = 0.215). Mean PUF score by smoking modality is shown in Table 4 and Figure 3.

**Table 4:** Mean PUF score by smoking modality. Levene's F(5,161) = 2.53, p = 0.031; Welch's F(5,17.3) = 1.59, p = 0.215 PUF: pelvic pain and urgency/frequency; SD: standard deviation Values are presented as n (% of current smokers) and mean PUF score (SD). Group means were compared using Welch's one-way analysis of variance because Levene's test showed unequal variances.

Smoking modality	n	%	Mean PUF score (SD)
Narghile	66	39.50%	7.92 (4.39)
Manufactured cigarettes	64	38.30%	8.37 (5.28)
Hand-rolled cigarettes	21	12.60%	10.35 (4.28)
Vape	8	4.80%	9.50 (4.10)
Pipe	4	2.40%	7.25 (1.25)
Cigars	4	2.40%	7.00 (2.70)
Total	167	100.00%	8.44 (4.68)

Daily cigarette/cigar quantity and PUF score

**Figure 4:**
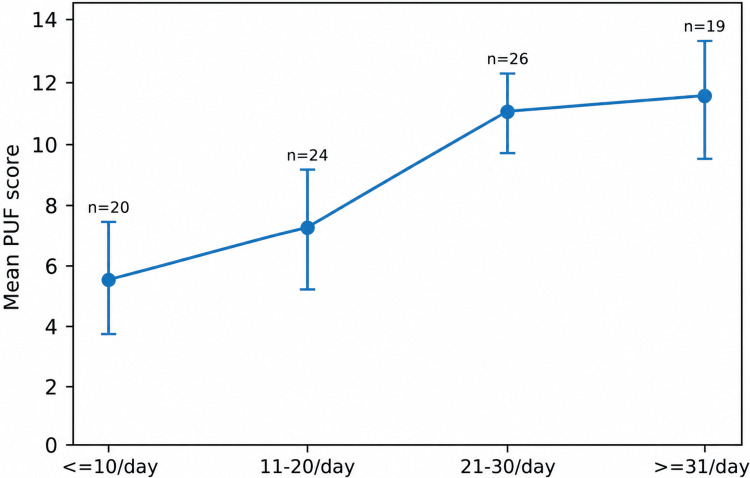
Mean PUF score by daily cigarette/cigar quantity. Bars show the mean pelvic pain and urgency/frequency (PUF) scores among cigarette/cigar users with available daily quantity data. Error bars represent 95% confidence intervals.

The daily quantity analysis was restricted to cigarette/cigar users and should not be generalized to narghile or vape exposure. Among 89 cigarette/cigar users with daily quantity data, the mean PUF score increased across daily quantity categories. The Levene test was not significant (F(3,85) = 1.39, p = 0.252), so standard one-way ANOVA was used (F(3,85) = 10.14, p < 0.001). Tukey post-hoc comparisons showed higher mean PUF scores in the 21-30/day and >=31/day groups compared with <=10/day (p < 0.001 and p < 0.001, respectively) and 11-20/day (p = 0.011 and p = 0.006, respectively). The <=10/day and 11-20/day groups did not differ significantly (p = 0.575), and the 21-30/day and >=31/day groups did not differ significantly (p = 0.972). Mean PUF score by daily cigarette/cigar quantity is summarized in Table 5 and Figure 4.

**Table 5:** Mean PUF score by daily cigarette/cigar quantity. PUF: Pelvic Pain and Urgency/Frequency Questionnaire; SD, standard deviation Levene's F(3,85) = 1.39, p = 0.252; one-way ANOVA F(3,85) = 10.14, p < 0.001. Analysis was restricted to cigarette/cigar users with available daily quantity data. Group means were compared using one-way analysis of variance. Tukey's honestly significant difference post hoc testing was used for pairwise comparisons because Levene's test was not significant.

Daily quantity	n (%)	Mean PUF score (SD)
≤10/day	20 (22.5%)	5.55 (3.99)
11-20/day	24 (27.0%)	7.17 (4.86)
21-30/day	26 (29.2%)	10.92 (3.23)
≥31/day	19 (21.3%)	11.47 (4.51)
Total	89 (100.0%)	8.82 (4.77)

Among the same 89 cigarette/cigar users with daily quantity data, the prevalence of PUF >9 increased across quantity categories, from 25.0% in the <=10/day group to 68.4% in the >=31/day group (χ²(3) = 11.38, p = 0.010). The association between daily cigarette/cigar quantity and higher-threshold PUF screening-positive symptom burden (PUF >9) is summarized in Table 6.

**Table 6:** Association between daily cigarette/cigar quantity and higher-threshold PUF screening-positive symptom burden (PUF >9). PUF: Pelvic Pain and Urgency/Frequency Questionnaire Analysis was restricted to cigarette/cigar users with available daily quantity data. Values are presented as n (%). Pearson's chi-square test was used.

Daily quantity	PUF >9 present	PUF >9 absent	p-value
<=10/day (n = 20)	5 (25.0%)	15 (75.0%)	
11-20/day (n = 24)	8 (33.3%)	16 (66.7%)	
21-30/day (n = 26)	16 (61.5%)	10 (38.5%)	
>=31/day (n = 19)	13 (68.4%)	6 (31.6%)	0.01
Total (n = 89)	42 (47.2%)	47 (52.8%)	

Caffeine intake and PUF screening-positive urinary symptom burden

**Figure 5:**
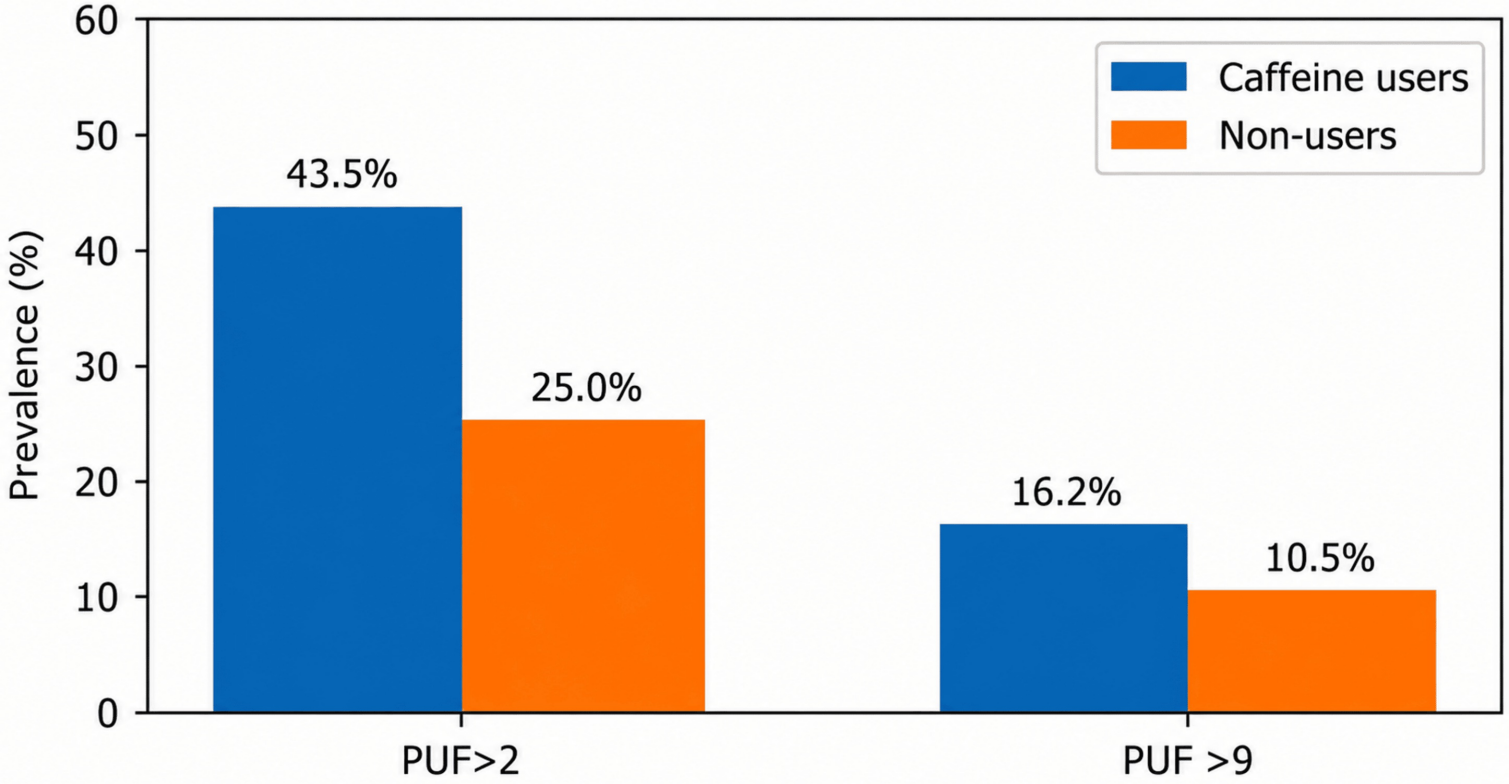
Prevalence of low-threshold PUF screening-positive urinary symptom burden (PUF >2) and higher-threshold PUF screening-positive symptom burden (PUF >9) by caffeine intake. Bars show the prevalence of pelvic pain and urgency/frequency (PUF) screening-positive urinary symptom burden (PUF >2) and higher-threshold PUF screening-positive symptom burden (PUF >9) among caffeine users and non-users.

Caffeine was consumed by 400 participants (84.0%). Low-threshold PUF screening-positive urinary symptom burden (PUF >2) was more common among caffeine users than non-users (43.5% vs 25.0%, χ²(1) = 9.06, p = 0.003). PUF >9 did not differ significantly by caffeine use (16.2% vs 10.5%, χ²(1) = 1.61, p = 0.204). Caffeine-related analyses are summarized in Table 7 and Figure 5.

**Table 7:** Association between caffeine intake and PUF screening-positive urinary symptom burden. CI: confidence interval; PR: prevalence ratio; PUF: Pelvic Pain and Urgency/Frequency Questionnaire Outcomes were defined as PUF screening-positive urinary symptom burden (PUF >2) and higher-threshold PUF screening-positive symptom burden (PUF >9). Pearson's chi-square tests were used.

Outcome	Caffeine users (n = 400)	Non-users (n = 76)	Crude prevalence ratio (95% CI)	p-value
Low-threshold PUF screening-positive urinary symptom burden (PUF >2)	174 (43.5%)	19 (25.0%)	1.74 (1.16-2.61)	0.003
PUF >9	65 (16.2%)	8 (10.5%)	1.54 (0.77-3.08)	0.204

Multivariable regression analyses

With current smoking modeled as the primary exposure and after adjustment for age, caffeine intake, sex, body mass index, and university type, current smoking showed the strongest independent association with both low-threshold PUF screening-positive urinary symptom burden (PUF >2) and higher-threshold PUF screening-positive symptom burden (PUF >9). Age was not independently associated with either outcome. Odds ratios are presented as association estimates and should not be interpreted as risk estimates, especially because low-threshold PUF screening-positive urinary symptom burden (PUF >2) was common. Multivariable logistic regression results are shown in Table 8.

**Table 8:** Multivariable logistic regression analysis of PUF screening-positive urinary symptom burden. BMI: body mass index; CI: confidence interval; OR: odds ratio; PUF: Pelvic Pain and Urgency/Frequency Questionnaire Models were adjusted for age, caffeine intake, sex, BMI, and university type. Reference categories were never-smoking status, no caffeine use, female sex, and public university. Odds ratios are presented as adjusted association estimates and should not be interpreted as risk estimates because the primary outcome was common.

Variable	Adjusted OR for PUF screening-positive urinary symptom burden (PUF >2) (95% CI)	p-value	Adjusted OR for PUF >9 (95% CI)	p-value
Smoking	21.56 (12.80-36.31)	<0.001	35.06 (14.33-85.77)	<0.001
Age, per year	1.02 (0.95-1.09)	0.59	1.03 (0.94-1.12)	0.54
Caffeine use	1.70 (0.85-3.41)	0.13	0.88 (0.34-2.27)	0.79
Male sex	0.72 (0.43-1.20)	0.21	0.61 (0.33-1.12)	0.11
Private university	0.84 (0.52-1.35)	0.48	1.70 (0.93-3.12)	0.08
BMI, per kg/m²	1.00 (0.94-1.07)	0.78	1.01 (0.93-1.09)	0.81

For low-threshold PUF screening-positive urinary symptom burden (PUF >2), model χ²(6) = 198.25, p < 0.001, and correct classification = 82.1%. For PUF >9, model χ²(6) = 129.73, p < 0.001, and correct classification = 85.5%. Reference categories were non-smoker, no caffeine use, female sex, and public university. Because only six non-smokers had PUF >9, the adjusted estimate for this outcome should be interpreted cautiously because of sparse events among non-smokers.

Adjusted prevalence ratios were also estimated using modified Poisson regression with robust error variance. These estimates were used to describe relative prevalence more directly than odds ratios for the common low-threshold PUF screening-positive urinary symptom burden (PUF >2) outcome. Smoking remained strongly associated with both outcomes, although the confidence interval for PUF >9 remained wide because of sparse events among non-smokers. Modified Poisson regression results are shown in Table 9.

**Table 9:** Adjusted prevalence ratios from modified Poisson regression with robust error variance. BMI: body mass index; CI: confidence interval; PR: prevalence ratio; PUF: Pelvic Pain and Urgency/Frequency Questionnaire Models were adjusted for age, caffeine intake, sex, BMI, and university type. Reference categories were never-smoking status, no caffeine use, female sex, and public university.

Variable	Adjusted PR for PUF screening-positive urinary symptom burden (PUF >2) (95% CI)	p-value	Adjusted PR for PUF >9 (95% CI)	p-value
Smoking	4.43 (3.49-5.63)	<0.001	18.12 (8.29-39.64)	<0.001
Age, per year	1.00 (0.98-1.03)	0.69	1.02 (0.96-1.08)	0.52
Caffeine use	1.18 (0.94-1.49)	0.15	0.91 (0.45-1.84)	0.79
Male sex	0.91 (0.76-1.09)	0.3 1	0.78 (0.48-1.26)	0.31
Private university	0.96 (0.81-1.13)	0.61	1.54 (0.91-2.60)	0.11
BMI, per kg/m²	1.00 (0.98-1.02)	0.82	1.01 (0.96-1.06)	0.77

Modality-stratified sensitivity analysis

An exploratory modality-stratified sensitivity analysis was performed using modified Poisson regression with robust error variance. The tobacco/nicotine use model included all current users and compared them with never-smoking non-users. The cigarette/cigar-only model included participants who reported only manufactured cigarettes, hand-rolled cigarettes, or cigars, without narghile, vape, or pipe use. The narghile-only model included participants who reported narghile use without other tobacco/nicotine modalities. Mixed-modality users were excluded from the cigarette/cigar-only and narghile-only models. Each model adjusted for age, caffeine intake, sex, body mass index, and university type. Results are shown in Table 10.

**Table 10:** Exploratory modality-stratified adjusted prevalence-ratio analysis. BMI: body mass index; CI: confidence interval; PR: prevalence ratio; PUF: Pelvic Pain and Urgency/Frequency Questionnaire All exposure categories were compared with never-smoking status. Adjusted prevalence ratios were estimated using modified Poisson regression with robust error variance. Models were adjusted for age, caffeine intake, sex, BMI, and university type. Mixed-modality users were included in the any tobacco/nicotine use model but excluded from the cigarette/cigar-only and narghile-only models.

Exposure definition	Outcome	Adjusted PR (95% CI)	p-value
Any tobacco/nicotine use	PUF >2	4.43 (3.49-5.63)	<0.001
Any tobacco/nicotine use	PUF >9	18.12 (8.29-39.64)	<0.001
Cigarette/cigar-only use	PUF >2	4.72 (3.54-6.29)	<0.001
Cigarette/cigar-only use	PUF >9	19.85 (8.67-45.45)	<0.001
Narghile-only use	PUF >2	4.08 (3.01-5.52)	<0.001
Narghile-only use	PUF >9	15.64 (6.71-36.45)	<0.001

In this exploratory sensitivity analysis, the association remained strong across any tobacco/nicotine use, cigarette/cigar-only use, and narghile-only use, with wide confidence intervals for PUF >9 reflecting sparse events among non-smokers.

Exploratory sex-stratified analyses

Exploratory sex-stratified analyses showed the same direction of association in males and females. For low-threshold PUF screening-positive urinary symptom burden (PUF >2), the crude prevalence ratio associated with smoking was 4.88 in males and 4.26 in females. For PUF >9, estimates were large in both sexes but unstable because only three male and three female non-smokers met the PUF >9 threshold. These exploratory sex-stratified findings are summarized in Table 11. 

**Table 11:** Exploratory sex-stratified association between smoking status and PUF screening-positive urinary symptom burden. CI: confidence interval; PR: prevalence ratio; PUF: Pelvic Pain and Urgency/Frequency Questionnaire Values are presented as events/total (%). Crude prevalence ratios compare current smokers with never-smoking nonsmokers within each sex stratum. Estimates for PUF >9 should be interpreted cautiously because events among nonsmokers were sparse.

Sex	Outcome	Smokers	Non-smokers	Crude PR (95% CI)
Male	PUF screening-positive urinary symptom burden (PUF >2)	77/94 (81.9%)	24/143 (16.8%)	4.88 (3.35-7.12)
Male	PUF >9	36/94 (38.3%)	3/143 (2.1%)	18.26 (5.79-57.58)
Female	PUF screening-positive urinary symptom burden (PUF >2)	60/73 (82.2%)	32/166 (19.3%)	4.26 (3.07-5.93)
Female	PUF >9	31/73 (42.5%)	3/166 (1.8%)	23.50 (7.42-74.41)

## Discussion

This cross-sectional study found a strong association between current smoking and questionnaire-based PUF screening-positive urinary symptom burden among Lebanese students and recent graduates. Low-threshold PUF screening-positive urinary symptom burden (PUF >2) was present in 82.0% of smokers compared with 18.1% of non-smokers. Higher-threshold PUF screening-positive symptom burden (PUF >9) was also markedly more common among smokers.

These findings are consistent with previous studies and reviews linking tobacco exposure to lower urinary tract symptoms and bladder pain syndrome [4,24-26]. In the adjusted models, smoking was the strongest predictor of both outcomes after accounting for age, caffeine intake, sex, body mass index, and university type. This supports the clinical value of asking about tobacco exposure, including narghile use, when evaluating young patients with urgency, frequency, nocturia, pelvic discomfort, or bladder pain.

The magnitude of association was large and should be interpreted cautiously. While a true relationship between tobacco exposure and urinary symptom burden is biologically plausible, the size of the estimates likely reflects a combination of the low, study-defined PUF >2 threshold, the self-selected online sample, volunteer bias related to social media recruitment, and residual confounding by unmeasured factors, such as urinary tract infection history, fluid intake, menstrual timing, sexual activity, stress, and gynecological conditions. These estimates should not be interpreted as showing that smoking alone explains the observed symptom excess. Because the outcome was common, prevalence ratios were emphasized alongside odds ratios. Adjusted prevalence-ratio models supported the same direction of association while giving a more interpretable relative-prevalence estimate for low-threshold PUF screening-positive urinary symptom burden (PUF >2). The adjusted estimate for PUF >9 remains sensitive to sparse data because only six non-smokers met this threshold.

A dose-response pattern was observed among cigarette/cigar users. Participants reporting higher daily cigarette/cigar quantity had higher mean PUF scores and a higher prevalence of PUF >9. The added exposure profile also showed that a meaningful proportion of smokers reported mixed-modality use and several years of cumulative exposure. Potential mechanisms include endothelial dysfunction, altered nitric oxide bioavailability, systemic inflammatory activation, urothelial injury, and platelet-activating factor signaling [13-17].

Narghile and manufactured cigarettes were the most common smoking modalities. Mean PUF scores were elevated across all smoking categories, but the study did not show statistically significant differences between modalities. This analysis was limited by small subgroups for vape, pipe, and cigar use. The safest interpretation is that current smoking overall was associated with urinary symptoms, while modality-specific differences require larger studies with more detailed exposure measurement.

The absence of statistically significant differences between modalities should not be interpreted as evidence that all tobacco/nicotine products have equivalent urinary effects. The vape, pipe, and cigar subgroups were small, and modality-specific analyses were underpowered. In addition, mixed-modality use may have diluted differences between individual products. Future studies should evaluate cigarettes, narghile, vaping products, cigars, and mixed exposure separately using larger samples and standardized dose metrics.

Caffeine intake was associated with low-threshold PUF screening-positive urinary symptom burden (PUF >2) in unadjusted analysis, but it was not independently associated with either low-threshold PUF screening-positive urinary symptom burden or higher-threshold PUF screening-positive symptom burden (PUF >9) after adjustment. This is compatible with prior evidence suggesting that caffeine may aggravate urgency or frequency in some patients, while its effect varies by dose, baseline bladder sensitivity, and other exposures [18-20]. In this dataset, smoking was the more consistent predictor. Because caffeine was coded only as use versus non-use, without dose, type, frequency, or timing, this crude measurement may have attenuated the observed caffeine-symptom association.

Clinical implications

Clinically, these findings suggest that tobacco exposure, including narghile and mixed-modality use, should be routinely assessed in young adults presenting with urinary urgency, frequency, nocturia, pelvic discomfort, or bladder pain. The findings do not establish causality, but they support incorporating smoking history into symptom assessment and counseling.

From a public health perspective, these findings support incorporating urinary symptom education into tobacco prevention and cessation programs targeting university-aged adults. Narghile use deserves specific attention because it was common in this population and may be perceived by young adults as less harmful than cigarette smoking [27]. Campus-based prevention programs could address urinary symptoms alongside the cardiovascular, respiratory, oncologic, and reproductive harms of tobacco and nicotine exposure [1]. Because the findings are questionnaire-based and cross-sectional, public health messaging should emphasize risk awareness and smoking reduction rather than implying that tobacco exposure directly causes clinically confirmed IC/BPS.

Strengths and limitations

This study focused on a young Lebanese population in which tobacco exposure, including narghile use, is common. It included a balanced sex distribution and a final sample size that exceeded the minimum requirement. The analysis addressed several clinically relevant exposure categories, including smoking status, smoking modality, smoking duration, mixed-modality use, daily cigarette/cigar quantity, estimated cigarette/cigar pack-years, narghile frequency, vape exposure, and caffeine intake.

The study also has important limitations. Its cross-sectional design prevents causal inference. Recruitment through social media may have introduced selection bias and volunteer bias, and the estimated response rate should be interpreted cautiously because the invitation denominator was derived from distribution lists rather than direct tracking of individual recipients. The questionnaire was administered only in English, and no translated Arabic PUF questionnaire was used, which may have affected comprehension and symptom reporting in some participants. Smoking and caffeine exposure were self-reported. Caffeine was analyzed only as use versus non-use, without dose, beverage type, timing, or frequency; this binary coding likely biased caffeine estimates toward the null. Several smoking modality subgroups were small, and the daily quantity analysis was restricted to cigarette/cigar users with available daily count data.

The study did not include urinalysis, urine culture, pelvic or urological examination, cystoscopy, urodynamic testing, or specialist confirmation. Symptom chronicity was not formally confirmed, which is important for clinical IC/BPS diagnosis. Additional potential confounders, including urinary tract infection history, sexual activity, sexually transmitted infection history, menstrual timing, gynecological disease, alcohol or energy drink intake, fluid intake, stress, constipation, and medication use, were not fully evaluated. Although exploratory sex-stratified analyses were performed, the study was not powered for definitive sex-specific inference, especially for PUF >9, because events among non-smokers were sparse. Future studies should evaluate sex-specific symptom patterns using clinically confirmed outcomes.

## Conclusions

Among Lebanese students and recent graduates aged 18-30 years, current smoking was strongly associated with low-threshold PUF screening-positive urinary symptom burden (PUF >2) and higher-threshold PUF screening-positive symptom burden (PUF >9). A dose-response pattern was observed among cigarette/cigar users, with higher daily consumption associated with higher mean PUF scores and higher PUF >9 prevalence. Caffeine was associated with low-threshold PUF screening-positive urinary symptom burden (PUF >2) in unadjusted analysis but was not independently associated with either outcome after adjustment. Because this was a cross-sectional questionnaire-based study without clinical confirmation, the findings should be interpreted as screening-based associations rather than evidence of clinically diagnosed IC/BPS or causal effects. Prospective studies with clinical evaluation are needed to clarify causality and determine whether tobacco/nicotine reduction improves urinary symptom burden.
